# Query enhancement through the practical application of ontology: the IEDB and OBI

**DOI:** 10.1186/2041-1480-4-S1-S6

**Published:** 2013-04-15

**Authors:** Randi Vita, James A Overton, Jason A Greenbaum, Alessandro Sette, Bjoern Peters

**Affiliations:** 1 Division of Vaccine Discovery, La Jolla Institute for Allergy and Immunology, 9420 Athena Circle, La Jolla, CA 92037, USA; 2 http://obi-ontology.org

## Abstract

Ontologies categorize entities, express relationships between them, and provide standardized definitions. Thus, they can be used to present and enforce the specific relationships between database components. The Immune Epitope Database (IEDB, http://www.iedb.org) utilizes the Ontology for Biomedical Investigations (OBI) and several additional ontologies to represent immune epitope mapping experiments. Here, we describe our experiences utilizing this representation in order to provide enhanced database search functionality. We applied a simple approach to incorporate the benefits of the information captured in a formal ontology directly into the user web interface, resulting in an improved user experience with minimal changes to the database itself. The integration is easy to maintain, provides standardized terms and definitions, and allows for subsumption queries. In addition to these immediate benefits, our long-term goal is to enable true semantic integration of data and knowledge in the biomedical domain. We describe our progress towards that goal and what we perceive as the main obstacles.

## 

The IEDB is a free resource that catalogs published experimental data regarding the recognition of epitopes by adaptive immune receptors. This is accomplished by a team of curator scientists who read relevant manuscripts and extract the data following a set of established guidelines [1]. In order to represent the data in a systematic and interoperable manner, the IEDB actively contributes to a number of Open Biological and Biomedical Ontology (OBO) projects [2]. The closest of these collaborations has been with the Ontology for Biomedical Investigations (OBI), an integrated ontology for the description of biological and clinical investigations [3]. OBI represents the experimental design, protocols, materials, and instruments used in biomedical investigations, the data generated and the type of analyses performed. Previously, we utilized OBI to model and export the immunological experiments described in the IEDB, allowing reasoning and driving database schema redesign [4,5]. Here we describe a deeper integration of IEDB assay types with OBI and discuss the many benefits for usability and querying. We close with a general discussion of the costs and benefits of integrating scientific data with ontologies, looking beyond the scope of IEDB, and offer our perspective on future prospects.

## Using ontologies to represent assays in the IEDB

**Figure 1:**
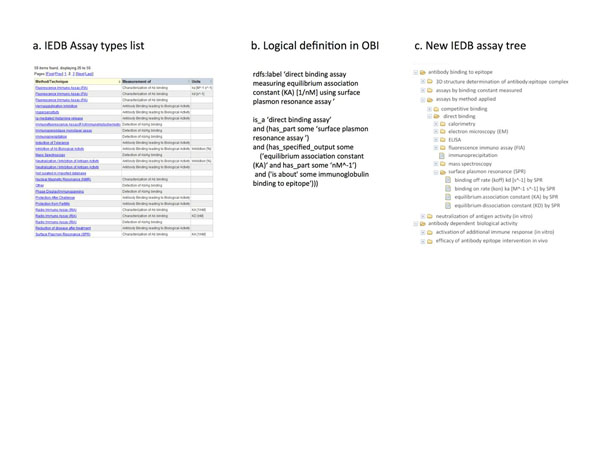
Conversion of IEDB assay types from a static list to terms in OBI used to generate new search interface utilizing a hierarchical tree.

IEDB curators collect a wide range of information about the experiments they curate. An accurate classification of the type of assays performed is crucial for a proper understanding of an experiment and the reported results. Initially, IEDB curators surveyed relevant epitope publications and itemized the types of assays that were encountered. Some assay types were straightforward and easily described, such as tetramer staining, while others were more complex, such as assays that assess the effect on disease development of immunizing mice with an epitope. A list of assay types was produced and used as a controlled vocabulary by curators adding new experiments to the database (Figure 1a).

This list-based approach was quite limited and did not capture the inherent similarities between different assay types. For example, the same method (e.g. surface plasmon resonance) may be used to measure different aspects of the same antibody: antigen binding event (e.g. equilibrium association and disassociation constants). Conversely, different methods such as ELISA and ELISPOT assays can be used to measure the same kind of biological event, such as IFN-g production by T cells. The different assay types utilized by the IEDB have relationships to each other that were not adequately described in a flat list of assays.

OBO projects such as OBI each provide a hierarchy of types (“terms”) as the primary axis of classification, and then enrich the hierarchy with additional relations. One branch of OBI is its hierarchy of assays. OBI defines ‘assay’ (OBI:0000070) as “A planned process with the objective to produce information about an evaluant” and gives the following logical definition (in part) using the Web Ontology Language (OWL) [6]:

IEDB assay types are more focused than this general type, involving specific biological events and measurement techniques, but each is a descendant of ‘assay’. Incorporating IEDB assays into OBI was an iterative and collaborative process, requiring careful consideration of term names, definitions, and relationships between terms in OBI and in other ontologies such as the Gene Ontology (GO) [7]. For example, ‘B cell epitope specific surface plasmon resonance (SPR) measuring KA [1/nM] assay’ (OBI:0001730) is logically defined as:

This logical definition refers to several terms from OBI, the Information Artifact Ontology [8], and other ontologies: two OBI assay types, ‘direct binding assay’ (OBI:0001591) and ‘surface plasmon resonance assay’ (OBI:0000923); a GO cellular component, ‘immunoglobulin complex’ (GO:0019814); a GO biological process, ‘immunoglobulin binding to epitope’ (OBI:0001702); several terms describing the output data, including ‘measurement datum’ (IAO:0000109), ‘equilibrium association constant (KA)’ (OBI:0001548), and ‘count per nanomolar’ (UO:0000284) from the Ontology of Units of Measurement (UO) [9]. The carefully defined relationships among terms provide a rich structure that supports multi-faceted classification and querying of the assay types and their instances.

For each assay type we identified the input materials, evaluants, outputs, and the information that was being produced (Figure 1b). Metadata such as a definition and an example are also required. The information produced by these immunological assays always relates either directly to a biological process or to some readout that is proxy for that process having occurred. Therefore, each assay was linked to a GO ‘biological process’ (GO:0008150) that the ‘information content entity’ generated by the assay ‘is_about’. Other external ontologies were also referenced as appropriate, such as UO when an assay has a specified readout of known units, e.g. “nM”. Term requests to outside ontologies were often required. The production of certain cytokines by T cells, including CCL4, CCL5, and CCL9, and antibody activities such as immune complex formation and neutralization of antigen are examples of terms that were added to GO based on our requests. In this way, the process of integration of our assay types into OBI enriched the content of several additional ontology projects.

The logical definitions of assays were often quite complex. However, despite the diversity of IEDB assay types, we discovered strong patterns among them. These “design patterns” allowed us to build a system of templates for efficiently describing specific assay types. The hierarchical organization of assay types also allowed much of the information about specific assay types to be inherited from their more general ancestor assay types. The following is part of a template used to generate term OBI:0001730 (mentioned above). Much of the information required for the logical definition is inherited from the more general assay types ‘direct binding assay’ (OBI:0001591) and ‘surface plasmon resonance assay’ (OBI:0000923).

• assay_type_id: 242

• label: B cell epitope specific surface plasmon resonance (SPR) measuring KA [1/nM] assay

• textual definition: surface plasmon resonance assay measuring epitope specific immunoglobulin equilibrium association constant (KA) in nM^-1

• IEDB alternative term full: surface plasmon resonance (SPR) measuring KA [1/nM]

• is a: direct binding assay (OBI:0001591)

• has part: surface plasmon resonance assay (OBI:0000923)

• measurement of: equilibrium association constant (KA) (OBI:0001548)

• has units: nM^∧^-1 (UO:0000284)

The Quick Term Template (QTT) method was used to generate OBI terms for the hundreds of assay types used in the IEDB [10]. This was easily accomplished by entering the metadata, such as definition, synonym, term editor, etc, associated with each assay type into the columns of Microsoft Excel spreadsheets. We then inserted these terms into OBI using a set of mapping expressions that formulate the logical definitions of each term. The Mapping Master plug-in of the Protégé ontology editor was used for this task [11], but the method is quite general can be implemented in many ways. Data can be drawn from a spreadsheet, relational database, text-file, etc. and inserted into a template using basic string-manipulation tools available in any programming language. The template itself can use one of several representations of OWL or RDF data, such as the Turtle syntax discussed below.

OBI has broad scope and a diversity of users. To ensure that each OBI term is well understood by a wide audience, the term labels are deliberately verbose. Within the context of IEDB and its website, where users expect to see information related to epitopes, these verbose labels are a drawback. We therefore created ‘IEDB alternative terms’ to provide the names commonly used by immunologists to describe assay types (Figure 1c). For example, the OBI term ‘ELISA of epitope specific granulocyte colony stimulating factor production by T cells’ is displayed by its shorter IEDB alternative term of ‘G-CSF release by ELISA’ because IEDB users expect all data to be epitope specific, are familiar with the abbreviation G-CSF, and know that all T cell assays will reflect a T cell response. Thus the benefits of a formal ontology (i.e., the standardized definitions, hierarchical tree, and term relationships) are provided while we avoid confusing end users with ontological jargon.

With the term design accomplished and the QTT templates developed, the task of migrating the hierarchical organization of assays in the ontology into IEDB and its search interface was straightforward. First, each of the existing IEDB assay types was mapped to an OBI identifier. The assay finder on the IEDB website is built using OWL API to read from the OBI.owl file [12]. Only descendants of the term ‘immune epitope assay’ are used in the assay finder, since this is the scope of the IEDB. The IEDB finder application is similar to tools provided by National Center for Biomedical Ontology (NCBO) Bioportal [13] which allow user interfaces to be populated using ontologies housed in Bioportal.

The overall effort involved in the implementation described here breaks down as follows. To replace the 244 assays in the IEDB with corresponding terms in OBI took a total of 4 man-months. The majority of this work went into analyzing how to precisely and formally model the assays in our list and their relations to one another. This involved reading papers in which they were used and talking to experts in the field. The technical steps of integration took 2 man-months, and were largely a one-off effort to enable our developers to read OWL files and convert the data to the formats required for the browsing and search interfaces of the IEDB website.

As the IEDB encounters new assay types in the literature, each is easily added to OBI utilizing the same QTT method. Once a new OBI.owl file is generated, the branch under ‘immune epitope assay’ simply replaces the existing one in use by the IEDB’s search interface. Updates are integrated into the build process, and require no human intervention.

## Immediate benefits from ontology integration

The conversion of the list of IEDB assays into an ontological hierarchy was time consuming, but in our opinion, the benefits have been significant and widespread, including: improved definitions, documentation, and understanding by curators and users; removal of duplicate assay types; improved curation accuracy; improved search by assay technique and biological event; and improved usability with hierarchical search.

Chief among these is improved understanding of the assay types by the IEDB curators and users. All IEDB assay types are now clearly documented, with textual and logical definitions. Having to clearly specify what makes two assay types different based upon the biological processes measured or the techniques applied has clarified curation rules. An exact definition allows a meaningful discussion of which type of assay is actually used in an investigation instead of arguing about labels for assays without definitions. Viewing groups of assays as siblings and seeing their parents also gives curators and users additional insight into the relationships between assays and improves understanding.

Selection from hierarchical structure is also better suited for curation than selection from a flat list of assays. If the description in a manuscript is not sufficient for a curator to decide which of two assays to pick, the curator can now select the parent class of those assays instead. For example, a manuscript may state that an epitope induced T cell degranulation, but not mention whether perforin or granzyme B was released. In the past, the curator would be forced to select an assay describing release of one of the two proteins. Using the new tree, the curator can select the parent class of ‘cytotoxic T cell degranulation’ instead, which more accurately reflects the information presented in the paper.

Automated reasoning over the ontology produces an inferred version of the hierarchy that allows for assays to appear in multiple locations. For example, all assays that use surface plasmon resonance will appear under the term ‘surface plasmon resonance assay’, regardless of what they measure (KA, KD, kon, etc.), while any surface plasmon resonance assay that also measures a KA will additionally appear under an organizational term representing assays measuring ‘equilibrium association constant (KA)’. The rich information in the logical definitions of the assay types supports this multi-faceted organization with no additional effort.

The hierarchical organization of assay types not only improves curation, but also enhances usability for browsing and search of IEDB. End users are now able to view all of the previously curated data in a hierarchically organized manner (Figure 1c). Formerly, end users were not able to select all assays that shared a parent, such as all assays that measure KA. Using the new tree, one may select all of a higher level of assay type, such as ELISA, or refine their criteria to a subset (ELISA with binding constant) or single assay type (ELISA with KD). Thus, hierarchical search significantly improves usability.

The enriched assay definitions also allow search options to include both what is measured (GO biological process) and how it is measured (OBI assay type). New content is being made available as each assay type now links, via the OBI identifier, to its metadata provided by OBI, giving users the option of viewing definitions and examples for the provided search terms.

Logical definitions have allowed us to remove duplicate assay types from the IEDB. Automated reasoners were able to infer from the logical definitions that several assay types were redundant. For example, because new assay types were added to the previous assay list as they were encountered in the literature, one assay measuring ‘chemokine (C-X-C motif) ligand 9 release’ and one measuring ‘MIG release’ were separately added to the list. The process of creating logical definitions for these assays based on GO biological processes followed by reasoning identified that the two assays were logically equivalent as the two terms are in fact referring to the same cytokine.

## Prospective benefits from ontology integration

A significant future benefit of integration of a formal ontology into the IEDB is the creation of rule-based validation. The logical restrictions and definitions of terms in OBI and other ontologies can be used to formulate curation rules. For instance, if an assay type is defined in OBI as requiring a virus as an input, then the curator must enter an input variable that is a virus. These rules can be extended to the external ontologies, such as GO. For example, if GO defines a certain cytokine as being produced only by CD4+ T cells, then an assay measuring that cytokine should not have CD8+ T cells curated as the effector cell.

Formal representation of all of the IEDB’s assay types within OBI has been one among a number of ways in which the IEDB builds on existing ontologies. Wherever possible, we are collaborating with existing projects and linking to other resources through ontological identifiers. We are in the process of integrating many of our classifications: cell types with the Cell Type Ontology [14]; tissue types with the Foundational Model of Anatomy [15]; diseases with the Human Disease Ontology [16]; organisms with NCBI Taxonomy [17]; proteins with the Protein Ontology [18]; and non-protein molecules from Chemical Entities of Biological Interest (ChEBI) [19]. One of the greatest benefits of these technologies is that they allow an enhanced range of queries across a variety of classification systems. For example, it becomes possible to use the GO biological process hierarchy to query for assays that measure ‘chemokine responses’ and distinguish them from other ‘cytokine responses’ even though the IEDB does not distinguish which cytokines are chemokines. As additional relevant ontologies are developed and imported, more sophisticated queries may be performed, providing new insights into the data of the IEDB.

**Figure 2:**
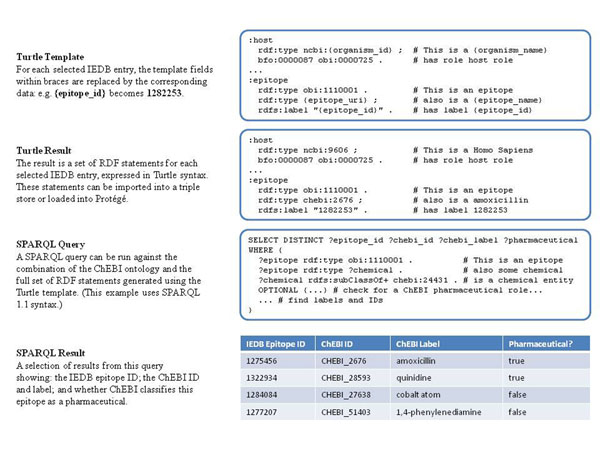
Export of IEDB data into RDF format makes SPARQL queries possible. This example demonstrates the ability to utilize the role branch of the ChEBI ontology to determine ‘pharmaceuticals’ with T cell responses in the IEDB. See http://ontology.iedb.org.

To enable queries of the IEDB data that take advantage of ontology term definitions, we translate selected IEDB data into RDF statements and then query the results (Figure 2) [20]. We use the Terse Triple Notation or “Turtle” syntax for representing RDF statements, and use a templating method similar to the QTT method discussed above [21]. We can then use SPARQL (a query language for RDF) to query the data set [22]. Figure 2 illustrates the use of ChEBI identifiers for epitope molecules in the IEDB. Using the information captured on the roles of different molecules in ChEBI, it becomes possible to ask which molecules are targeted by T cell immune responses and are also used as pharmaceuticals (a ChEBI:role). Similarly, using the Vaccine Ontology, we can ask what pathogens for which a human vaccine exists have been characterized in terms of their T cell and B cell responses after natural infection [23].

The URL for a SPARQL query endpoint can be found at http://ontology.iedb.org. We envision that the SPARQL endpoint will initially be used as a proof of concept by the relatively few users familiar with this technology. It allows us and others to test whether biological queries requested by end users can indeed be better answered by this approach. Queries that are deemed useful will be integrated into the standard IEDB web interface, which does not require our end users to have any knowledge of the ontology or these linked data technologies.

## Conclusions and perspective

The IEDB has been pursuing integration with ontologies for seven years. We have been fortunate to find many ontology projects relevant to the data housed by the IEDB, and continue to actively seek out further collaborations. We have tried to learn from others working on similar problems, such as (among many others) the eagle-i discovery system [24], NIF [25], and EuPathDB [26]. Our investments of time and resources have resulted in a number of immediate benefits for the IEDB described above, but the long term promise of seamless data integration across different projects has not yet been realized.

The problems faced by the IEDB are general ones, and widely shared. Data need to be classified. The literature contains a diversity of terminology. Rather than using an ad hoc list of terms, there are benefits to collaborating on shared standards. Policies such as standardized identifiers and IRIs, and technologies such as RDF and OWL, make it easier to name terms, annotate them, and encode information in rich networks of terms. The QTT method makes it easier to add large sets of similar terms to ontologies. Turtle templates make it easier to move data from tabular-form into RDF graphs. Projects using shared best practices will find it easier to merge RDF graphs into broad networks of linked data.

While progress has been made over the years, it has often been slow. We believe that there are three primary reasons for the slow progress: The speed (or lack thereof) of community based ontology development, gaps in tools and best practices, and a lack of examples for advanced ontology utilization in database projects. To a certain extent, the slow speed of community based ontology development is unavoidable. It is difficult to find a consensus on how knowledge should be represented in a way that satisfies experts from different domains. However, these problems are amplified by the lack of direct funding for ontology development. OBO projects are modeled after open-source software projects and, like most open-source projects; development is done on a volunteer basis. Contributors come from various scientific projects, such as IEDB, and have specific goals to accomplish for their home projects. The result is often a lack of documentation, “technical debts” such as poor testing and release procedures, and a lack of shared focus. The combination of scientific expertise and specialized technical skills required to create and maintain an ontology project is relatively rare. The learning curve is relatively steep. Ontologies such as OBI are built by small, changing groups of active volunteers, within a larger circle of contributors who have a long-term interest but lack the time or the skills to play a larger role.

However, the benefits of open ontology projects are easy to share, requiring only a little knowledge and no special skills. Ontologies and linked data projects benefit from “network effects”, where the advantage of joining a large network is much greater than joining a small one. Investments in this sort of informatics infrastructure for science may yield widespread savings, as individual scientific projects are saved from having to reinvent systems of classification, and as the value of linked data increases. We would like to see greater support for community ontology projects from granting agencies.

We would also like to see the costs of development for ontology project lowered by further development of shared best practices and supporting tools. The OBO Foundry provides a number of policies and best practices that facilitate interoperability between ontologies and the scientific projects that use them. We have found these policies useful and would like to see them expanded in scope, not because they are perfect, but rather because having any standard (and a mechanism for changing it) is preferable to having none. There are also several tools and services that facilitate ontology development, such as Protégé and Bioportal. Investments in the continued development of such tools for building high-quality ontologies should reduce the barriers to entry for scientists, thus increasing participation in ontology development and speeding progress.

Finally, we believe that providing working examples of ontology based data integration across large data repositories is necessary to demonstrate the feasibility and usefulness of the approach. Our own efforts are aimed at providing one such example. We hope that eventually this will lead to a tipping point, when the benefits of advanced ontology use will be obvious while the costs for new projects to follow these practices will have dropped substantially based on the ability to re-use existing ontologies, standards and tools.

## Competing interests

The authors declare that they have no competing interests.

## Authors’ contributions

RV participated in ontology modeling, created/edited ontology terms, and wrote and edited the manuscript and figures. JAO designed the Turtle template, performed the export of data and SPARQL queries, and wrote and edited the manuscript and figures. JAG participated in ontology modeling, created/edited ontology terms, participated in data export, and wrote and edited the manuscript. AS evaluated and improved the ontology modeling as a domain expert. BP conceived and oversaw the entire project, participated in ontology modeling, created/edited ontology terms, and wrote and edited the manuscript. All authors read and approved the final manuscript.

## Declarations

All fees and funding for this article were paid by the National Institutes of Health contract HHSN2272201200010C.

This article has been published as part of *Journal of Biomedical Semantics* Volume 4 Supplement 1, 2013: Proceedings of the Bio-Ontologies Special Interest Group 2012. The full contents of the supplement are available online at http://www.jbiomedsem.com/supplements/4/S1
